# Hystrosalpingo-foam sonography with ExEm and lidocaine-based foam gel for detecting tubal occlusion: a diagnostic test accuracy systematic review and meta-analysis

**DOI:** 10.1007/s00404-025-08105-4

**Published:** 2025-07-07

**Authors:** Mohamed K. A. Genedy, Reem Shalata, Marwa El-Difrawy, Abdelrahman A. Almetwally, Rahma Sameh Shaheen

**Affiliations:** 1 Faculty of Medicine, Cairo University, Cairo, Egypt; 2 Faculty of Medicine, Benha University, Benha, Egypt

## Dear sir,

The fallopian tube is essential for natural conception, and its obstruction or damage induces infertility, accounting for 30–45% of cases [1]. Typically managed by assisted reproductive techniques [2, 3]. Testing tubal patency in infertile females, especially those with risk factors, is essential, as per guidelines [2, 4]. Laparoscopy dye test (LDT), the gold standard for accurate assessment, involves financial burden and risks, including anesthesia exposure and organ injury [5]. Hysterosalpingography (HSG), a less invasive option, involves contrast medium injection through the cervix and X-ray imaging [3]. Despite being less invasive and good diagnostic performance in general, it had a *27.5%* discordance in bilateral occlusion and a *high* false positive rate in unilateral occlusion, most likely due to spasms caused by pain and insufficient filling [3]. In addition it comes with radiation exposure, delayed results in oil-based contrasts and other hazards [5].

Hysterosalpingo-foam sonography (HyFoSy) was developed in 2010 for diagnosing tubal occlusion by Exalto N and Emanuel MH [6]. It recently gained attention and demonstrated high accuracy, as well as lower pain rates but lacked accessibility. Ludwin et al. introduced lidocaine-based gel in 2017 as part of Hysterosalpingo-Lidocaine-Foam Sonography with Power Doppler (HyLiFoSy-PD) [7]. HyLiFoSy-PD is easy to implement in medical centers globally due to its low cost and wide availability of lidocaine gel [7]. The “flaming tube” sign, which both practitioner and patient can easily identify, is promising and has potential to be the first line in future; however, currently literature on it is scarce [7]. It’s theorized to have pain reduction effects, and accordingly reduction of tubal spasms and false positive rates [7].

HyFoSy as a diagnostic test for tubal occlusion has been previously investigated in two meta-analyses by Cassiman et al. (2024) and Melcer et al. (2020) [8, 9]. However, both have notable limitations that we adresses. Melcer et al. (2020) included studies with LDT or HSG as reference test, in females undergoing fertility work up and device sterilization confirmation [9]. It showed an exaggerated diagnostic metrics of *99%* sensitivity and *91%* specificity with high heterogeneity. This is likely due to including HSG imperfections and difference in populations. Cassiman et al. 2024 compared HyFoSy with other tubal patency tests, focusing on tolerance, safety, and efficacy [8]. The study was not a DTA oriented and didn’t assess sensitivity or specificity along with other diagnostic metrics; the study assessed the agreement of HyFoSy and LDT via dichotomous analysis [8]. In addition, they defined bilaterally patent tubes only as patent, counting unilaterally patent tubes as occluded [8]. The study concluded that HyFoSy outperforms HyCoSy with a *94%* agreement rate [8].

On 19th October 2024, we conducted a comprehensive search across five databases (PubMed, Web of Science Core Collection, Scopus, Cochrane Library, and Embase) using terms related to HyFoSy, ultrasound, tubal occlusion, and infertility (Table S1). We have included five diagnostic test accuracy (DTA) studies comparing HyFoSy and LDT in infertile or subfertile females of childbearing age with primary or secondary infertility enrolled for fertility work-up (Fig. S1). We avoided studies with HSG reference standard due to its imperfections [3]. Study quality was using QUADAS-2 [10], and data extraction was performed by the authors using standardized sheets, covering study and population characteristics, diagnostic accuracy outcomes, and complications. The meta-analysis was conducted on the Meta-DiSc 2.0 [11], using a bivariate random-effect models with a 95% confidence interval (CI) with 1428 tubes, and 5 inconclusive results excluded, of which 545 were diseased and 883 were non-diseased (Table S3).

Almost all five studies [5, 12–14] used ExEm gel for sonography, except for Sharaf et al. (2022) used lidocaine gel [15]. Participants were 775 females, typically between 27 and 34 years old. The number of tubes and participants varied widely, ranging from 40 to 868 (Table S2). There was an overall low risk of bias and low applicability concerns in the included studies [5, 12–15] (Fig. S2).

**Fig. 1:**
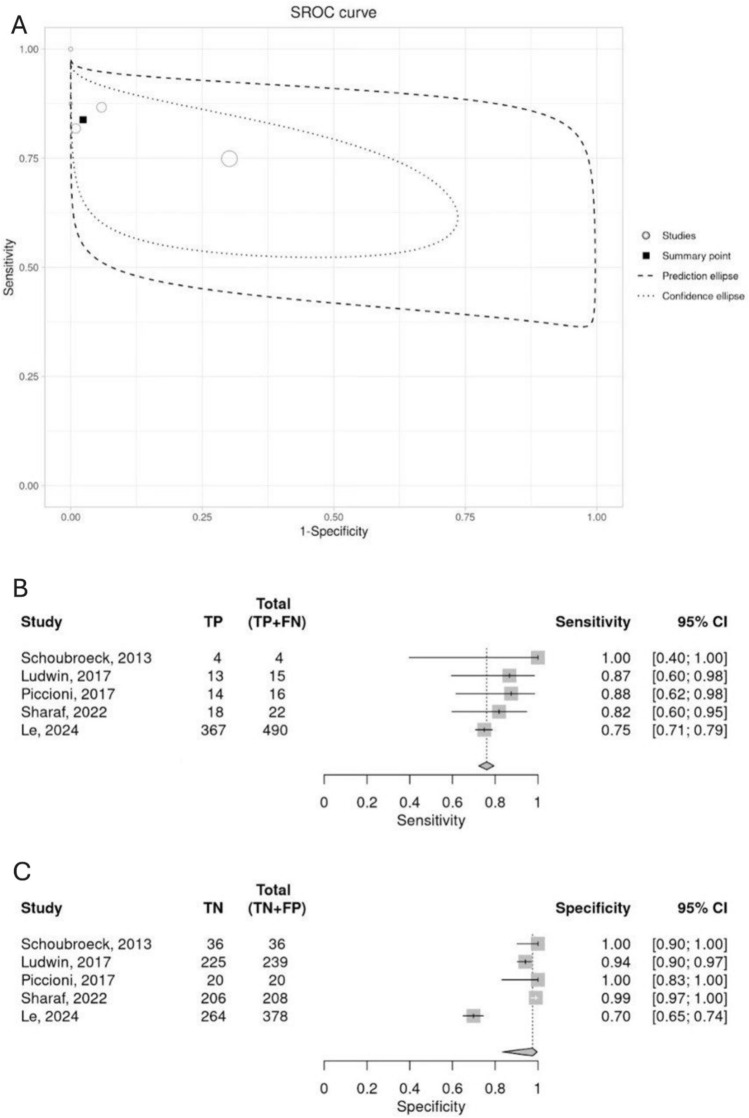
**A** Summary points on ROC plot for sensitivity and specificity. **B** Sensitivity forest plot. **C** Specificity forest plot

In the forest plots shown in Fig. 1B, C, most studies showed high diagnostic accuracy individually, reporting *specificity ranging from 0.94 to 1.00 and sensitivity 0.87 to 1.00*, with some variability inspected*.* Le 2024 [5] them most recent study with largest sample size, stands out with *lower specificity (0.70, 95% CI: 0.65–0.74) and sensitivity (0.75, 95% CI: 0.71–0.79)*, which rises concerns about HyFoSy applicability in real-world settings.

In the ROC plot shown in Fig. 1A, the curve shows high sensitivity and specificity, with most studies clustered toward the top-left corner, suggesting strong diagnostic accuracy [5, 12–15]. This is evident with pooled diagnostic values as high as *0.87 (95% CI: 0.74–0.94)* in sensitivity and *0.97 (95% CI: 0.84–0.99)* specificity, as well as excellent diagnostic odds ratio of *193.73 (95% CI: 20.51–1829.36),* low false positive rate of *0.03 (95% CI: 0.006– 0.16)*, positive and negative likelihood ratio of *25.74 (95% CI: 4.66–142.26)* and *0.13 (95% CI: 0.06–0.29)*, respectively; suggesting both excellent diagnostic performance and reliable exclusion of disease (Table S5).

The bivariate *I*^2^ is *0%,* and the sensitivity’s logit variance shows low heterogeneity of *0.266*. However, the specificity log variance has a substantially higher variance of *2.486* (Table S6), which explains the broad prediction ellipse, particularly along the specificity axis (Fig. 1A).

In ExEm and Lidocaine subgroup analysis, both show strong diagnostic accuracy, with ExEm achieving *0.86 (95% CI: 0.70–0.94)* sensitivity and *0.95 (95% CI: 0.76–0.99)* specificity*,* while Lidocaine has slightly higher sensitivity *(0.9, 95% CI: 0.62–0.98%)* and specificity *(0.98, 95% CI: 0.75–0.99)*. The diagnostic odds ratio is *128.41 (95% CI: 11.65–1415.28)* for ExEm and *519.70 (95% CI: 8.76 –30,846.09)* for Lidocaine, indicating high diagnostic power, though wide confidence intervals suggest some uncertainty. Both foam gels have low false positive rate *(ExEm: 0.05, 95% CI: 0.007–0.24, Lidocaine: 0.02, 95% CI: 0.001–0.25)* and negative likelihood ratio *(ExEm: 0.15, 95% CI: 0.0 6–0.35, Lidocaine: 0.1, 95% CI: 0.02–0.49)*, supporting reliable exclusion of disease (Table S7). However, HyLiFoSy-PD might be better in terms of pain and cost reduction. Hence, we recommend further research to explore it as a potential first line test for tubal occlusion detection.

## Supplementary Information

Below is the link to the electronic supplementary material.

Supplementary file1 (DOCX 1096 KB)

## Data Availability

The data that supports the findings of this study are available within the manuscript.
