# Supplementary material for: Hystrosalpingo-foam sonography with ExEm and lidocaine-based foam gel for detecting tubal occlusion: a diagnostic test accuracy systematic review and meta-analysis
Source: Arch Gynecol Obstet. 2025 Jul 7;312(5):1383–6. doi: 10.1007/s00404-025-08105-4 (PMC12589321; doi:10.1007/s00404-025-08105-4)
Supplement: Supplementary file 1 — Supplementary file1 (DOCX 1096 KB) [file 404_2025_8105_MOESM1_ESM.docx]

**Supplementary 1.**

**Diagnostic Test Accuracy of Hystrosalpingo-Foam Sonography for Detecting Tubal Occlusion: A Systematic Review and Meta-Analysis**

**Running Title.** Accuracy of Hystrosalpingo-Foam Sonography

**Authors.** Mohamed K. A. Genedy1*, Reem Shalata1, Marwa El-Difrawy1, Abdelrahman A. Almetwally1, Rahma Sameh Shaheen2

**Affiliations.**

1. Faculty of Medicine, Cairo University, Cairo, Egypt

2. Faculty of Medicine, Benha University, Benha, Egypt.

*** Corresponding author:**

Mohamed K. A. Genedy

Email: mo7med.kamel2017@gmail.com

ORCID: 0009-0009-8234-1557

Faculty of Medicine, Cairo University, Cairo, Egypt

Mobile: (+20)1102465107)

Postal address: 11th District 6th of October Post Office (Postal Code: 15555)

**Author’s information:**

Mohamed K. A. Genedy

Email: mo7med.kamel2017@gmail.com

ORCID: 0009-0009-8234-1557

Faculty of Medicine, Cairo University, Cairo, Egypt.

Reem Shalata

Email: reemshalata58@gmail.com

ORCID: 0000-0002-0830-1348

Faculty of Medicine, Cairo University, Cairo, Egypt.

Marwa El-Difrawy

Email: eldifrawymarwa@gmail.com

ORCID: 0009-0006-6788-8907

Faculty of Medicine, Cairo University, Cairo, Egypt.

Abdelrahman A. Almetwally

Email: drabdelrahmanshehata3@gmail.com

ORCID: 0009-0009-3948-0957

Faculty of Medicine, Cairo University, Cairo, Egypt.

Rahma Sameh Shaheen

Email: rahma193226@fmed.bu.edu.eg

ORCID: 0000-0001-5200-6138

Faculty of Medicine, Benha University, Benha, Egypt.

**Content:**

**Figures and Tables Legends.**

Fig.S1. PRISMA flow chart.

Fig.S2. Summary of QUADAS 2 quality assessment.

Table S1. Details of search strategy for each database.

Table S2. Summary of study characteristics.

Table S3. Summary of Studies Diagnostic Metrics.

Table S4. Summary of failure, and pain rates.

Table S5. Summary statistics.

Table S6. Summary of Heterogeneity.

Table S7. Subgroup analysis.


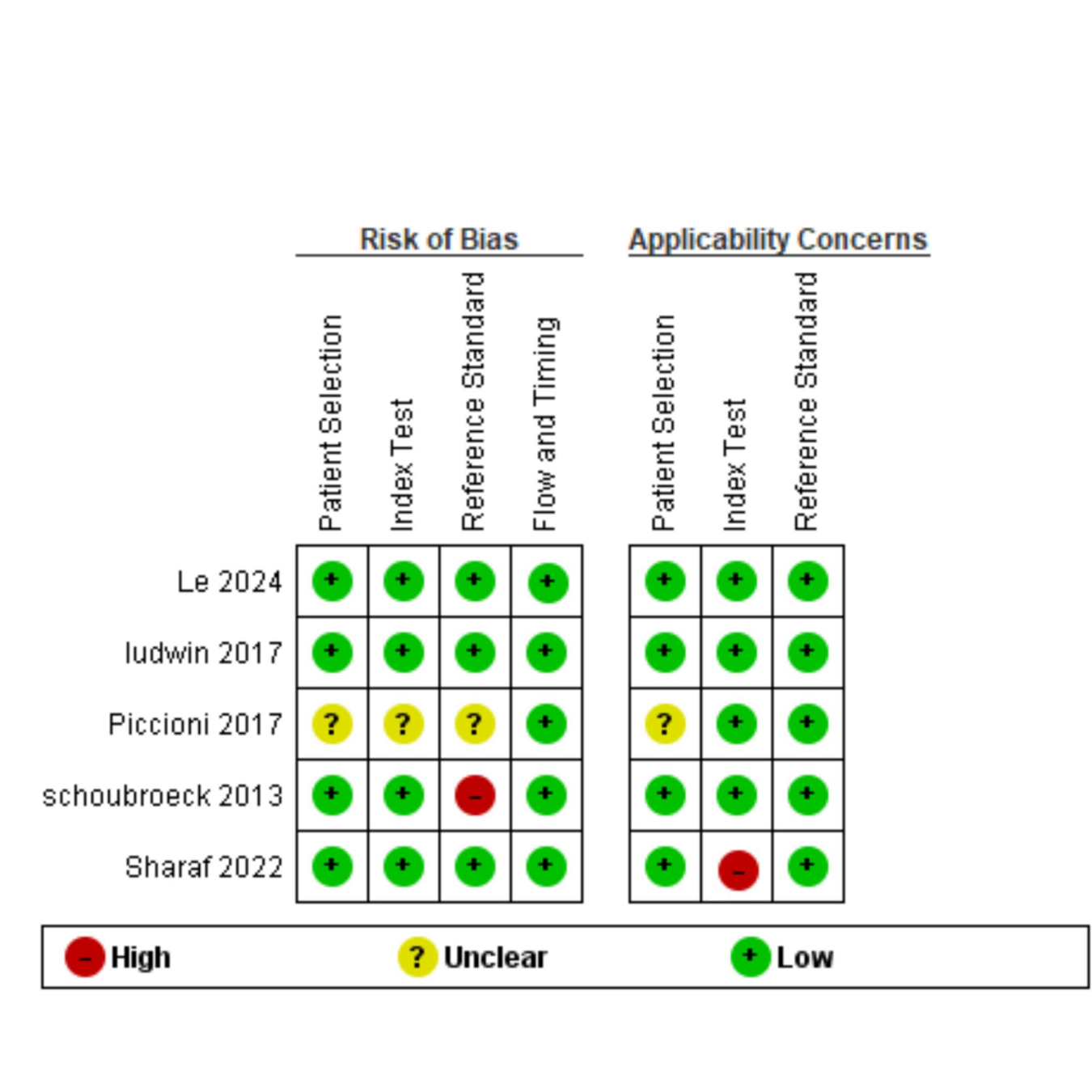
Fig.S2. Summary of QUADAS 2 quality assessment.


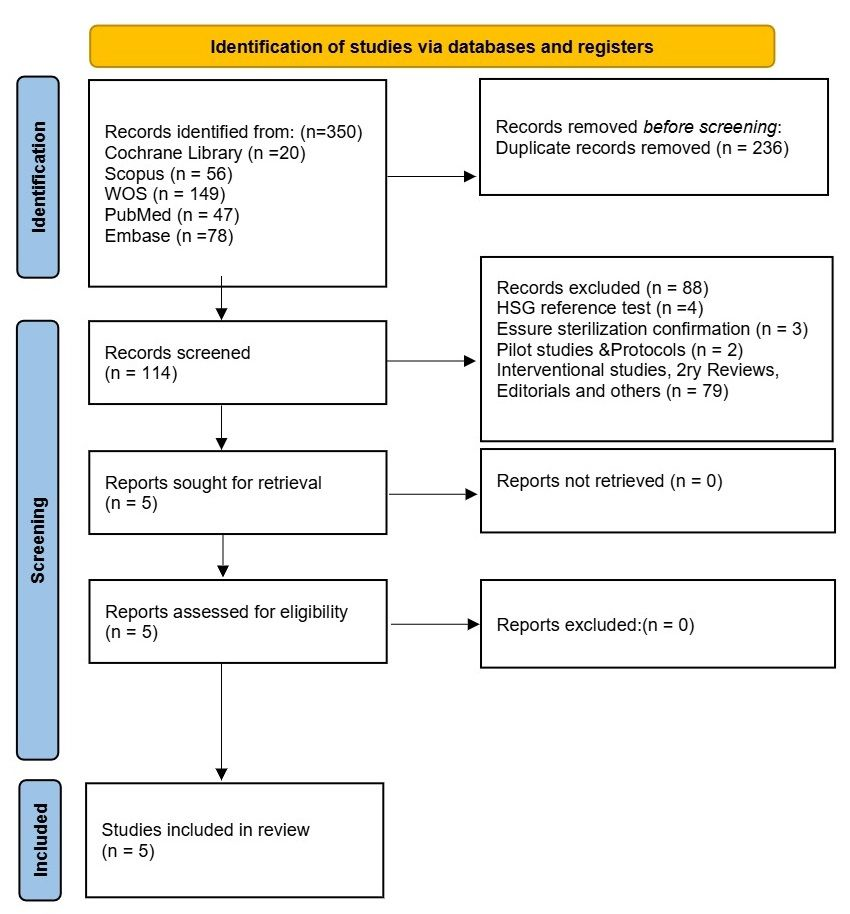
Fig.S1. PRISMA flow chart.

| **Database** | **Search Terms** | **Search Field** | **Search Results** |
| --- | --- | --- | --- |
| **PubMed** | ("HyFoSy" OR "Foam") AND ("Hysterosalpingography" OR "sonography" OR "ultrasound" OR "ultrasonography") AND "Tube" AND ("patent" OR "patency" OR "occlusion" OR "obstruction" OR "blockade" OR "closure") | All Field | 47 |
| **Cochrane** | ("HyFoSy" OR "Foam") AND ("Hysterosalpingography" OR "sonography" OR "ultrasound" OR "ultrasonography") AND "Tube" AND ("patent" OR "patency" OR "occlusion" OR "obstruction" OR "blockade" OR "closure") | All Field | 20 |
| **WOS** | ("HyFoSy" OR "Foam") AND ("Hysterosalpingography" OR "sonography" OR "ultrasound" OR "ultrasonography") AND "Tube" AND ("patent" OR "patency" OR "occlusion" OR "obstruction" OR "blockade" OR "closure") | Topic | 149 |
| **Scopus** | ("HyFoSy" OR "Foam") AND ("Hysterosalpingography" OR "sonography" OR "ultrasound" OR "ultrasonography") AND "Tube" AND ("patent" OR "patency" OR "occlusion" OR "obstruction" OR "blockade" OR "closure") | Title, Abstract, Keywords | 56 |
| **Embase** | ("HyFoSy" OR "Foam") AND ("Hysterosalpingography" OR "sonography" OR "ultrasound" OR "ultrasonography") AND "Tube" AND ("patent" OR "patency" OR "occlusion" OR "obstruction" OR "blockade" OR "closure") | All Field | 78 |

Table S1. Details of search strategy for each database.

| **Study ID** | **Number Of Tubes** | **Number Of Populations** | **Age**  **Mean (SD)** | **Fertility Status** | **Type Of Fertility N (%)** | | **Sonography** | **Foam Gel** |
| --- | --- | --- | --- | --- | --- | --- | --- | --- |
|  |  |  |  |  | **Primary** | **Secondary** |  |  |
| Schoubroeck et al. 2013 ^24^ | 40 | 20 | 31 ± 13.56 | infertile | 14 (70%) | 6 (30%) | 3D vaginal | ExEm gel |
| Ludwin et al. 2017 ^22^ | 259 | 132 | 32.3 ± 4.3 | infertile/subfertile | 103 (78%) | 29 (22%) | 2D/3D | ExEm gel |
| Piccioni et al. 2017 ^7^ | 74 | 37 | 34 ± 0.471 | infertile | 37 (100%) | 0 | NR | ExEm gel |
| Sharaf et al. 2022 ^23^ | 230 | 139 | 27 ± 5.24 | infertile/subfertile | 66 (57.4%), | 49 (42.6%) | 2D | Lidocaine gel |
| Le et al. 2024 ^5^ | 868 | 447 | 33.3±5.2 | infertile/subfertile | 242 (53.2%) | 213 (46.8%) | 2D | ExEm gel |

Table S2. Summary of Study Characteristics.

2D/3D: Two /Three-Dimensional, N: Number, NR: Not Reported, SD: Standard Deviatioland

| **Study ID** | **TP** | **FP** | **FN** | **TN** | **Inconclusive Results** | **Sensitivity** | **Specificity** | **PPV** | **NPV** | **Accuracy** |
| --- | --- | --- | --- | --- | --- | --- | --- | --- | --- | --- |
| Schoubroeck et al. 2013 ^24^ | 4 | 0 | 0 | 36 | 0 | 100.00% | 100.00% | 100.00% | 100.00% | 100.00% |
| Ludwin et al. 2017 ^22^ | 13 | 14 | 2 | 225 | 5 | 86.67% | 94.14% | 48.15% | 99.12% | 93.70% |
| Piccioni et al. 2017 ^7^ | 14 | 0 | 2 | 20 | 0 | 87.50% | 100.00% | 100.00% | 90.91% | 94.44% |
| Sharaf et al. 2022 ^23^ | 18 | 4 | 2 | 206 | 0 | 90.00% | 98.10% | 81.82% | 99.04% | 97.39% |
| Le et al. 2024 ^5^ | 367 | 114 | 123 | 264 | 0 | 74.90% | 69.84% | 76.30% | 68.22% | 72.70% |

Table S3. Summary of Studies Diagnostic Metrics.

True positive (TP), false positive (FP), false negative (FN), true negative (TN), positive Predictive Value (PPV), negative Predictive Value (NPV)

| **Study ID** | **Test** | **Failure Rate (Event/Total)** | **Reasons For Failure** | **Number of Participants Reporting Pain (Event/Total)** | **Pain Scale** | **Notes** |
| --- | --- | --- | --- | --- | --- | --- |
| Ludwin et al. 2017 ^22^ | HyFoSy | 4/261 | Unbearable pain during catheter insertion (N = 3) | Severe: 2/261 | NRS | Efferalgan codeine tablet was administered one hour before ultrasound examinations, including HyFoSy and HyCoSy procedures. |
|  |  |  | Unbearable pain during saline Infusion (N = 1) |  |  |  |
| Sharaf et al. 2022 ^23^ | HyLiFoSy | 7/122 | Severe pain during the introduction of the speculum (N = 7) | Mild: 91/115, Moderate: 24/115 | VRS | Azithromycin was given as antibiotic prophylaxis (one day before, the day of, and one day after the procedure). The specific NSAID used was not mentioned. |
| Le et al. 2024 ^5^ | HyFoSy | 6/455 | Cervical Stenosis (N = 1) | Mild:129/449, Moderate: 115/449, Sever: 13/449 | VAS | NR |
|  |  |  | Foam Reflux (N = 3) |  |  |  |
|  |  |  | Extreme Pain (N = 1) |  |  |  |
|  |  |  | Moderate Pain and Discomfort (N =1) |  |  |  |

Table S4. Summary of failure, and pain rates.

NR: not reported, NRS: Numerical Rating Scale, VAS: visual analogue scale, VRS: Verbal Rating Scale.

| **Diagnostic metric** | **Estimate [95% CI]** |
| --- | --- |
| Sensitivity | 0.872 [0.741, 0.941] |
| Specificity | 0.966 [0.837, 0.994] |
| DOR | 193.725 [20.515, 1829.361] |
| LR+ | 25.744 [4.659, 142.26] |
| LR- | 0.133 [0.061, 0.29] |
| FPR | 0.034 [0.006, 0.163] |

Table S5. Summary statistics.

Cl: Confidence Interval, DOR: Diagnostic Odds Ratio, FPR: False Positive Rate, LR +/-: Positive and Negative Likelihood Ratio.

| **Heterogeneity metrics** | **Estimate** |
| --- | --- |
| Var logit(sen) | 0.266 |
| Var logit(spe) | 2.486 |
| Bivariate I2 | 0 |

Table S6. Summary of Heterogeneity.

Var logit(spe/sen): variance log its specificity and sensitivity.

| **Diagnostic metric** | **Estimate Exem [95% CI]** | **Estimate Lidocaine [95% CI]** |
| --- | --- | --- |
| Sensitivity | 0.86 [0.701 , 0.942] | 0.901 [0.619 , 0.981] |
| Specificity | 0.954 [0.763 , 0.993] | 0.983 [0.751 , 0.999] |
| DOR | 128.417 [11.652 , 1415.279] | 519.706 [8.756 , 30846.086] |
| LR+ | 18.787 [3.001 , 117.607] | 52.436 [2.658 , 1034.601] |
| LR- | 0.146 [0.062 , 0.348] | 0.101 [0.021 , 0.489] |
| FPR | 0.046 [0.007 , 0.237] | 0.017 [0.001 , 0.249] |

Table S7. Subgroup analysis.

Cl: Confidence Interval, DOR: Diagnostic Odds Ratio, FPR: False Positive Rate, LR +/-: Positive and Negative Likelihood Ratio.
